# MicroRNA Cargo in Wharton’s Jelly MSC Small Extracellular Vesicles: Key Functionality to In Vitro Prevention and Treatment of Premature White Matter Injury

**DOI:** 10.1007/s12015-023-10595-1

**Published:** 2023-07-31

**Authors:** Vera Tscherrig, Sophie Cottagnoud, Valérie Haesler, Patricia Renz, Daniel Surbek, Andreina Schoeberlein, Marianne Simone Joerger-Messerli

**Affiliations:** 1 Department of Obstetrics and Feto-maternal Medicine, University Women’s Hospital, Inselspital, Bern University Hospital, Bern, Switzerland; 2 Graduate School for Cellular and Biomedical Sciences (GCB), University of Bern, Bern, Switzerland; 3 Department for BioMedical Research (DBMR), University of Bern, Bern, Switzerland

**Keywords:** Premature white matter injury, Wharton’s jelly mesenchymal stromal cells, small extracellular vesicles, microRNA

## Abstract

Preterm birth is the leading cause of childhood morbidity and mortality and can result in white matter injury (WMI), leading to long-term neurological disabilities with global health burden. Mesenchymal stromal cell-derived small extracellular vesicles (MSC-sEV) are a promising therapeutic agent for treating perinatal neurological injury. They carry microRNAs (miRNAs) predicted to be involved in the onset of premature WMI. We hypothesize that miRNAs have a key function in the beneficial effects of MSC-sEV. We isolated MSC from umbilical cord tissue, the Wharton’s jelly (WJ), and purified small extracellular vesicles (sEV) from WJ-MSC culture supernatant by ultracentrifugation and size exclusion chromatography. The miRNA content was quantified by real-time polymerase chain reaction. A luciferase gene assay validated silencing of *TP53* and *TAOK1*, which we previously identified as predicted target genes of MSC-sEV miRNAs by Next Generation Sequencing and pathway enrichment analysis. The impact of sEV miRNAs on oligodendroglial maturation and neuronal apoptosis was evaluated using an in vitro oxygen-glucose deprivation model (OGD/R) by knocking-down *DROSHA* in WJ-MSC, which initiates miRNA processing. WJ-MSC-sEV contained miRNAs involved in WMI, namely hsa-miR-22-3p, hsa-miR-21-5p, hsa-miR-27b-3p, and the hsa-let-7 family. The luciferase assay strongly indicated an inhibitory effect of sEV miRNAs on the gene expression of *TP53* and *TAOK1*. Small EV initiated oligodendrocyte maturation and reduced OGD/R-mediated neuronal apoptosis. Knocking-down *DROSHA* in WJ-MSC reduced the expression of sEV miRNAs and led to the loss of their beneficial effects. Our in vitro study strongly indicates the key function of miRNAs in the therapeutic potential of WJ-MSC-sEV in premature WMI.

**Supplementary Information:**

The online version contains supplementary material available at 10.1007/s12015-023-10595-1.

## Introduction

According to the World Health Organization (WHO), approximately 15 million neonates are born preterm yearly, meaning that every tenth birth is premature. Despite progress in medical care, around 1 million neonates die every year due to premature birth [1]. Premature-born neonates are at high risk for neurological injuries such as white matter injury (WMI), often leading to long-term neurological disabilities [2]. Although the number of newborns affected by the more severe cystic periventricular leukomalacia (PVL) has declined, milder forms of the disease, such as non-cystic WMI, persist. In non-cystic WMI, normal white and gray matter development is disrupted, leading to neurobehavioral and neurodevelopmental impairments. Hence, the reduction of mortality from premature birth does not correlate with a reduction in short- and long-term neurological disabilities [2]. Neurological consequences include impaired cognition, attention, behaviour, and socialization, leading to a significant public health burden. Neonates are at high risk of developing WMI when born before 32 weeks of gestation [2, 3].

The pathophysiology of WMI is complex. It involves inflammatory and hypoxic/ischemic (HI) insults triggering excitotoxicity and oxidative stress [3]. The free radicals generated by HI are particularly harmful to the precursor cells maturing into the myelin-forming oligodendrocytes [3]. In addition, caspase-dependent cell death affects neuronal cells and oligodendrocyte precursors and participates in WMI pathogenesis [3]. Until now, there is no cure for WMI.

Progress in research has shown that mesenchymal stromal cells (MSC), and especially their secreted extracellular vesicles (EV), have therapeutic potential in neonatal hypoxic-ischemic brain injury [4–13]. Initially, EV were considered a by-product for the disposal of unused proteins [14, 15]. However, over the past decades, the role of EV in intercellular communication by transferring proteins, lipids, DNA, mRNA, and non-coding RNA to distal cells was recognized [16–18]. As a non-living agent, EV have several advantages over whole-cell therapeutics. They can be stored at -80 °C for several months and distributed efficiently [19]. Furthermore, EV have lower spleen and liver toxicity, are less immunogenic, and efficiently cross biological barriers upon intranasal delivery [4–9, 20]. Three classical subtypes of EV exist, namely apoptotic bodies, ectosomes, and exosomes, which can be distinguished according to their size, biogenesis, and cellular origins [21, 22]. However, the nature of biogenesis of isolated EV is rarely known. Thus, the most recent Minimal Information for Studies of Extracellular Vesicles (“MISEV”) guidelines proposed to standardize the nomenclature according to the size and to distinguish between small EV (sEV) that have a diameter of < 200 nm and large EV that have a diameter > 200 nm [21]. To comply with MISEV guidelines, we use the operational term sEV referring to the size of our vesicles in this study. Small EV are composed of a phospholipid bilayer membrane and contain proteins, lipids, and coding and non-coding RNAs, such as microRNAs (miRNAs), reflecting their donor cells’ biological properties [23]. The cargo of sEV has been studied intensively, and mature miRNAs turned out to be a key component of sEV in physiology and disease [24, 25]. In contrast to free circulating miRNAs [26], miRNAs coated with the sEV membrane are protected from being degraded by RNase [27] allowing a safe shuttle of functional miRNAs from cell to cell [16].

In a preclinical rat model of premature brain injury, we have shown the therapeutic potential of intranasally applied sEV derived from MSC isolated from the umbilical cord connective tissue, called Wharton’s jelly (WJ) [7, 8]. More specifically, WJ-MSC-derived sEV downregulated microglia-mediated neuroinflammation by reducing the production of Tnfα and Il1β [7, 8]. Our group previously identified the sEV miRNA cargo as a promising mediator of these beneficial effects in experimental WMI. Highly abundant miRNAs were identified in the WJ-MSC-sEV samples using Next Generation Sequencing Analysis [28]. These miRNAs are interfering with MAPK/ERK and Notch signalling cascades [28]. In particular, many of the miRNAs enriched in sEV were predicted to target the genes encoding the tumor suppressor gene *TP53* and the Thousand And One Amino Acid Protein Kinase 1 (*TAOK1*) [28]. *TP53* encodes a protein with a crucial role in inflammation and apoptosis induction [28–30]. TAOK1 is involved in apoptosis and inflammation by acting upstream of p38 MAPK and c-Jun N-terminal kinase (JNK) 1/2/3 and being part of the Hippo pathway [28, 31]. The potential of the miRNA cargo of sEV for brain injury therapy has been investigated in a variety of studies [32–34].

To induce translational repression and mRNA degradation, miRNAs have to bind to the microRNA response elements (MREs) in the 3’ untranslated region (3’UTR) of the target mRNA [35]. The biogenesis of mature miRNAs is temporally and spatially tightly controlled and can be classified into canonical and non-canonical pathways [36]. Thereby, miRNAs are predominantly processed by the canonical pathway, involving the cleavage of the primary (pri)-miRNAs into precursor (pre)-miRNAs by the Microprocessor complex, encompassing the RNA binding protein DiGeorge Syndrome Critical Region 8 (DGCR8) and the Class 2 ribonuclease III Drosha in the nucleus. Pre-miRNAs are exported into the cytoplasma by the exportin 5 (XPO5)/RanGTP complex and cleaved by the ribonuclease III Dicer into mature miRNAs [37, 38]. A *DROSHA* knock-out results in the loss of mature miRNAs mainly generated by the canonical pathway [36, 39].

Based on these recent findings, we hypothesize that miRNAs enriched in WJ-MSC-derived sEV have a crucial function in the observed beneficial effects of the sEV. Thus, we aimed to investigate the functionality of the miRNAs of WJ-MSC-derived sEV in vitro. First, the functionality of the miRNAs was analysed in a luciferase reporter gene assay. Two vectors containing the 3’UTR of TP53 or TAOK1, both predicted target genes of the miRNAs in WJ-MSC-sEV, were used to assess the ability of the miRNAs to regulate the gene expression by binding to miRNA-binding sites. Moreover, as premature white matter injury is characterized by impaired oligodendrocyte maturation and increased neuronal death, we compared the effects of naïve sEV and sEV derived from *DROSHA* knock-down WJ-MSC on the oligodendrocyte precursor cell line MO3.13 and the anti-apoptotic effects on the neuroblastoma cell line N2a, previously exposed to oxygen-glucose deprivation/reoxygenation to trigger neuronal cell death.

## Materials and Methods

### Isolation of sEV Derived from Human Wharton’s Jelly Mesenchymal Stromal Cells (WJ-MSC)

The study was approved by the Ethics Committee of the Canton of Bern. Human umbilical cords from healthy term deliveries (gestational age ≥ 37 weeks) were collected after informed consent. From the connective tissue of the umbilical cord, the Wharton’s jelly, mesenchymal stromal cells (WJ-MSC) were isolated using enzymatic digestion as previously described [40]. The cells were cultured in Dulbecco’s modified Eagle’s medium (DMEM)/F12 (Thermo Fisher Scientific Inc., Waltham, MA, USA) supplemented with 10% fetal bovine serum (FBS) (Thermo Fisher Scientific Inc.), 2 mmol/l GlutaMAX™ (Thermo Fisher Scientific Inc.), 100 units/ml penicillin and 100 µg/ml streptomycin (21331046, Thermo Fisher Scientific Inc.). At passages 4 to 5, cells at 80% confluency were prepared for sEV-isolation as previously described [40]. Briefly, WJ-MSC were washed twice with 1x phosphate-buffered saline (PBS), and the medium was replaced with serum-free medium containing DMEM/F12, 2 mmol/L GlutaMAX™, 100 units/ml penicillin and 100 µg/ml streptomycin for 36 – 40 h. Cell culture supernatant was collected, and sEV were isolated by serial ultracentrifugation (UC), according to the protocol of Théry et al. [41], followed by size-exclusion chromatography (SEC) using an IZON automatic fraction collector (IZON Science Ltd., Addington, Christchurch, New Zealand) with PBS as collection buffer. The protein and RNA contents of the sEV fractions were measured using a NanoVue Plus™ spectrophotometer (Biochrom, Holliston, MA, USA). The sEV-containing SEC fractions were combined and stored at − 80 °C until use.

### Characterization of WJ-MSC

#### Immunocytochemistry

Cultured WJ-MSC were visualized with bright field microscopy (Leica DFC 300 FX, Leica Microsystems, Wetzlar, Germany). For the analysis of cell surface markers, WJ-MSC were seeded in 2-well chamber slides at a density of 2000 cells/cm^2^. Upon adherence, cells were fixed with 4% paraformaldehyde (PFA) in PBS, blocked with PBS containing 1% bovine serum albumin (BSA; Merck KGaA, Darmstadt, Germany) and stained with fluorescein isothiocyanate (FITC)-conjugated mouse monoclonal antibodies against CD90 (11-0909-42, Thermo Fisher Scientific Inc.) and CD45 (550539, BD Biosciences, Franklin Lakes, NJ, USA), and with a polyclonal rabbit antibody against CD73 (550256, BD Biosciences Inc.). The antibodies were diluted 1:100 in blocking buffer and incubated overnight. For the detection of the unconjugated antibody against CD73, the cells were incubated the next day with an Alexa-Fluor 594-conjugated anti-mouse IgG antibody (1:1000, a11005, Thermo Fisher Scientific Inc.) for 1 h at room temperature. Stained WJ-MSC were analysed using a fluorescent microscope (Leica CTR 6000).

#### Flow Cytometric Analysis

WJ-MSC were analysed by flow cytometry for cell surface markers as previously described [7]. The cells were stained with an adenomatous polyposis coli protein-conjugated (APC) mouse monoclonal antibody against CD105 (562408, BD Biosciences Inc.) and FITC-conjugated mouse monoclonal antibodies against CD90 (SM1170F, OriGene Technologies, Inc., Rockville, MD, USA), CD45 (555482, BD Biosciences Inc.), CD34 (555821, BD Biosciences Inc.), CD14 (MAB1219F, Merck KGaA), and human leukocyte antigen–antigen D related (HLA-DR) (555811, BD Biosciences Inc.), as well as the unconjugated antibodies against CD73 (550256, BD Biosciences Inc.) and CD19 (FCMAB184F, clone HD37, Merck KGaA). An Alexa-Fluor 594-conjugated anti-mouse IgG antibody (a11005, Thermo Fisher Scientific Inc.) was used to detect CD73 and CD19 antibodies. All antibodies were diluted in PBS containing 1% FBS to their respective working concentrations (Supplementary Table 1) and incubated with WJ-MSC for 15 min at 4 °C. At least 10’000 events were acquired on a LSR II flow cytometer (BD Biosciences Inc.) and data were analysed using FlowJo™ v10.8 Software (BD Biosciences Inc.).

### Characterization of WJ-MSC-sEV

Multiple techniques were used to determine the characteristics of sEV, as suggested by the Minimal Information for Studies of Extracellular Vesicles (MISEV) 2018 guidelines [21].

#### Negative-Staining Electron Microscopy

The shape of WJ-MSC-sEV was analysed with negative-staining electron microscopy. For imaging of negatively stained samples, 5 µl of the vesicle suspension was adsorbed on glow-discharged and carbon-coated 400 mesh copper grids (Plano, Wetzlar, Germany) for 0.5–1 min. After washing them three times by dipping them in pure water, grids were stained with 2% uranyl acetate in water (Electron Microscopy Science, Hatfield, PA, USA) for 45 s. The excess fluid was removed by gently pushing them sideways to filter paper. Samples were examined with a transmission electron microscope (Tecnai Spirit, FEI Company, Hillsboro, OR, USA) at 80 kV, equipped with a digital camera (Veleta, Olympus, Münster, Germany).

#### Nanoparticle Tracking Analysis

A ZetaView® x20 (Particle Metrix GmbH, Inning am Ammersee, Germany) has been used to analyse the particle concentration, size, and zeta potential of sEV. The sEV samples were diluted in PBS, and 600–1000 µl of the diluted sample was added to the ZetaView® x20. Eleven positions throughout the sample were analysed according to their size. A mean size was generated by the software included with the system.

Classical micro-electrophoresis was used to measure the zeta potential. The ZetaView® x20 was flushed with filtered de-ionized water (ddH_2_O) before the analysis to prevent pH changes. The sEV samples were diluted in ddH_2_O, and 1 ml of the diluted sample was loaded to the ZetaView® x20. Eleven positions through the sample were analysed for their zeta potential. The mean zeta potential was calculated by the software included with the system.

#### Western Blot Analysis

Small EV (5 µg total protein) were separated by sodiumdodecylsulfate–polyacrylamide gel electrophoresis (SDS-PAGE) on a 4–20% gradient gel (4561094, Bio-Rad, Hercules, CA, USA), transferred to PVDF membranes (IB401002, Thermo Fisher Scientific Inc.), and blocked with 5% non-fat dry milk dissolved in Tris-buffered saline containing 0.05% Tween 20 (Merck KGaA; TBST). The following proteins were analysed: CD63 (1:500, PA5-92370, Thermo Fisher Scientific Inc.), CD81 (1:1000, PA5-79003, Thermo Fisher Scientific Inc.), CD73 (1:1000, ab175396, Abcam, Cambridge, United Kingdom), Calnexin (1:1000, ab22595, Abcam) and GM130 (1:1000, MA5-35107, Thermo Fisher Scientific Inc.). Horseradish peroxidase-coupled donkey anti-rabbit antibody (NA9340, Merck KGaA) was used as secondary antibody in a 1:1000 dilution. The binding of the antibodies was detected using the chemiluminescent Amersham ECL Prime Western Blotting Detection Reagent (GEHERPN2232, Cytiva, Marlborough, MA, USA) on a C-DiGit Blot Scanner (LI-COR Biosciences, Lincoln, NE, USA).

#### Imaging Flow Cytometric Analysis

In total, 5 × 10^6^–5 × 10^7^ sEV per ml were diluted 1:200 in an antibody staining solution for 1 h at room temperature. The following antibodies have been used: APC-conjugated anti-CD63 (A15712, Thermo Fisher Scientific Inc.), phycoerythrin (PE)-conjugated anti-CD81 (A15781, Thermo Fisher Scientific Inc.), and FITC-conjugated anti-CD9 (ab18241, Abcam). The samples were then further diluted at either 1:50 or 1:100 in PBS. Measurements have been performed on an ImageStream X Mark II Imaging Flow Cytometer (Luminex Corporation, Austin, Texas, USA). Samples were analysed using the FlowJo™ v10.8 Software (BD Biosciences Inc.).

#### Adenosine Assay

To further analyse the WJ-MSC-sEV’s capability to transform ATP to adenosine via the CD73-pathway, 1 µg WJ-MSC-sEV was incubated for 1 h with 100 µM ATP. The adenosine production was then measured with a fluorometric adenosine assay (ab211094, Abcam) according to the manufacturer’s protocol and detected with a fluorescent plate reader (TECAN Spark 10 M, Tecan Trading AG, Männedorf, Switzerland) with excitation and emission at 535 and 587 nm, respectively. PBS was used as a control to exclude the background from the phosphate in the PBS.

### Stimulation of Murine Microglial BV-2 Cells with Lipopolysaccharide (LPS) and Co-Culture with WJ-MSC-sEV

The semi-adherent mouse immortalized microglial cell line BV-2 (ATL03001) was purchased from Banca Biologica Cell Factory, Genova, Italy, and expanded in Roswell Park Memorial Institute (RPMI) 1640 (21870084, Thermo Fisher Scientific Inc.) containing 10% FBS, 2 mmol/L GlutaMAX™, 100 units/ml penicillin and 100 mg/ml streptomycin. BV-2 cells were detached from culture plates by mechanical vibrations and flushing with PBS. 18’000 cells/cm2 were seeded on a 6-well plate and left to grow overnight. The next day, cells were incubated with 100 ng/ml LPS for 1 h, either with or without adding 1 µg/ml of WJ-MSC-sEV. Subsequently, RNA and protein were isolated. Untreated BV-2 cells were used as a control.

### Luciferase Assay Using Human Embryonic Kidney (HEK293T) Cells

By means of the web server TargetScan [42] and the microRNA Data Integration Portal (mirDIP (version: 5.3.0.1)) [43], we identified miRNA-binding sites in the 3’ untranslated regions (3’UTR) of *TP53* and *TAOK1*, respectively. These miRNAs were highly abundant in our WJ-MSC-sEV [28]. To analyse the direct interaction with, and thus the post-transcriptional regulation of, *TP53* and *TAOK1* by WJ-MSC-sEV miRNAs we used a luciferase assay with pMirTarget reporter constructs containing the 3’UTR of either human *TP53* (pMirTarget-*TP53*) (SC214918, OriGene Technologie Inc.) or human *TAOK1* (pMirTarget-*TAOK1*) (SC214715, OriGene Technologies Inc.) upstream of the firefly luciferase reporter gene.

#### Bacterial Transformation and Plasmid DNA Preparation

For the transformation, 1 µl of DNA was mixed gently into 100 µl of JM109 competent cells (L200, Promega, Madison, Wisconsin, USA). The competent cell/DNA mixture was incubated on ice for 20–30 min, followed by a heat shock at 42 °C for 45 s. The tubes were then immediately put on ice for 2 min. 900 µl of SOC medium (15544034, Thermo Fisher Scientific, Inc.) was added, and the mixture was incubated at 37 °C for 45 min on a shaking incubator. Thereafter, 100 µl of the transformation mix were plated onto a 10 cm Luria Broth (LB) agar plate containing 25 µg/ml kanamycin (11815024, Thermo Fisher Scientific, Inc.) and incubated at 37 °C overnight. The next day, single bacterial clones were picked from the agar plates, added to liquid LB (10855001, Thermo Fisher Scientific, Inc.), mixed with 25 µg/ml kanamycin, and incubated at 37 °C on a shaker overnight. Vector DNA was isolated on the following day using the Plasmid MiniPrep kit (D4209, Zymo Research, Irvine, CA, USA) according to manufacturer’s instructions.

#### HEK293T Cells Transfection and Luciferase Assay

Human embryonic kidney (HEK293T) cells were obtained from American Type Culture Collection (CRL-11268,  ATCC, Manassas, Virginia, USA). For the luciferase assay, 20’000 HEK293T cells per well were seeded into a white flat-bottom 96-well plate (Greiner Bio-One, Kremsmünster, Austria). The next day, the HEK293T cells were transfected with 50 ng of vector DNA diluted in 50 µl of Opti-MEM I Reduced Serum Media without serum (31985070, Thermo Fisher Scientific Inc.) and 0.5 µl of Lipofectamine 2000 Reagent (11668027, Thermo Fisher Scientific Inc.). Either 1 µg/ml WJ-MSC-sEV or the same volume of PBS was added. Upon culture for 48 h, the luciferase signal was measured using the Dual-Glo Luciferase Assay System (E2920, Promega Corporation, Madison, WI, USA) according to the manufacturer’s instructions. The luciferase signal was normalized to the vector-internal signal of the red fluorescent protein. Luminescence and fluorescence were measured with a fluorescent plate reader (TECAN Spark 10 M, Tecan Trading AG).

#### Modulation of *TP53* 3’UTR

The functionality of the sEV miRNAs was further verified by modifying the 3’UTR region of pMirTarget-*TP53* mRNA with the QuikChange Lightning Site-Directed Mutagenesis Kit (210518, Agilent Technologies Inc., Santa Clara, CA, USA) according to the manufacturer’s protocol. Point mutations of the miRNAs binding sites for hsa-miR-22-3p and hsa-let-7-5p, previously identified using TargetScan [42], and mirDIP [43] (see above) were created with mutagenic primers designed with the QuikChange Primer Design Program. Thermal cycling was performed to sequentially introduce the point mutations into the 3’UTR (Supplementary Table 2) for each binding site. The amplification products were digested with 2 µl of Dpn I restriction enzyme, included in the kit, at 37 °C for 5 min. The modified vectors were then transformed into XL10-Gold ultracompetent cells provided with the kit. The cells were spread on LB-agar plates containing 100 µg/ml ampicillin and incubated at 37 °C for > 16 h. Single bacterial clones were collected to isolate the mutated plasmids (pMirTarget-*TP53mut*) with the Plasmid MiniPrep kit (D4209, Zymo Research Inc.).

### Knock-down of *DROSHA* in WJ-MSC

A small interfering RNA (siRNA) against the human *DROSHA* gene (1299001, Thermo Fisher Scientific Inc.) was used to knock-down *DROSHA* expression in WJ-MSC. WJ-MSC were cultured in DMEM/F12 containing 10% FBS, 2 mmol/l GlutaMAX™, 100 units/ml penicillin and 100 µg/ml streptomycin. When the cells reached 60% confluency, they were transfected with *DROSHA* siRNA as follows: per T150 flask of WJ-MSC, 300 pmol *DROSHA* siRNA and 50 µl RNAiMAX reagent (13778075, Thermo Fisher Scientific Inc.) were mixed in 5 ml OptiMEM ™ Reduced Serum Medium (31985070, Thermo Fisher Scientific Inc.). The medium was changed to serum-free DMEM/F12 for sEV isolation 72 h post-transfection. Small EV (*DROSHA* k.d sEV) were isolated from the conditioned medium and analysed as described above.

### Culture of Oligodendrocytic Hybrid Cell Line MO3.13 with WJ-MSC-Derived sEV

The human oligodendrocytic hybrid cell line MO3.13 was expanded in DMEM (61965059, Thermo Fisher Scientific Inc.) supplemented with 10% FBS, 2 mmol/l GlutaMAX™, 100 units/ml penicillin and 100 mg/ml streptomycin. For the culture with WJ-MSC-sEV, MO3.13 cells were treated as previously described with small modifications [28]. In brief, cells were seeded at a density of 3’500 cells/cm^2^ and left to adhere overnight. The next day, 1 µg/ml of either WJ-MSC-sEV or *DROSHA* k.d sEV was added to the cells and cultured for either 6 h, 24 h, 48 h or 3 days. As a control, the cells were cultured without WJ-MSC-sEV. Subsequently, the cells’ mRNA expression of Neuroblastoma RAS viral oncogene homolog (*NRAS*), Mitogen-activated protein kinase 1 (*MAPK1*), and Notch homolog 1 (*NOTCH1*), and the phosphorylation of ERK1/2 and the cleavage of Notch1 were analysed to monitor oligodendrocyte maturation induction as described in 2.11 and 2.12 (Table 1).

**Table 1:** qPCR primer sequences used for gene expression and miRNA expression (F: forward primer, R: reverse primer)

Gene	Description	Assay ID/Primers and probe sequence
*GAPDH*	Glyceraldehyde-3-phosphate dehydrogenase	F: GCTCCTCCTGTTCGACAGTCA R: ACCTTCCCCATGGTGTCTGA Probe: CGTCGCCAGCCGAGCCACA
*NRAS*	Neuroblastoma RAS viral oncogene homolog	HS00180035_m1
*MAPK1*	Mitogen-activated protein kinase 1	Hs01046830_m1
*NOTCH1*	Notch homolog 1	F: TGCATGATGCCTACATTTCAAGA R: TTCAGTATTATGTAGTTGTTCGTTGGTTATAC Probe: TGGTTCTGGAGGGACC
*Caspase 3*		Mm01195085_m1
*Trp53*		Mm01731290_g1
*Taok1*		Mm00522816_m1
*Tnfa*		Mm00443258_m1

### Culture of Mouse Neuroblastoma Cell Line Neuro2a (N2a) with Oxygen-Glucose Deprivation (OGD)

The mouse neuroblastoma cell line Neuro2a (N2a) was purchased from American Type Culture Collection (CCL-131™, ATCC). The cells were expanded in DMEM (61965059, Thermo Fisher Scientific Inc.) containing 10% FBS, 100 units/ml penicillin, and 100 mg/ml streptomycin (N2a expansion medium).

Oxygen-glucose deprivation and reoxygenation (OGD/R) was performed as previously described [40]. Briefly, N2a cells were seeded in 100 mm cell culture dishes at a density of 9’000 cells/cm^2^ and left to adhere overnight. The next day, the N2a expansion medium was replaced by glucose-free DMEM (11966025, Thermo Fisher Scientific Inc.), supplemented with 10% FBS, 100 units/ml penicillin, and 100 mg/ml streptomycin, and incubated for 6 h at 37 °C in 1% O_2_ and 5% CO_2_ (hypoxia). After 6 h, the medium was replaced with N2a expansion medium, 1 µg/ml of either WJ-MSC-sEV or *DROSHA* k.d sEV was added, and the cells were returned to a normoxic incubator for 48 h. The cells were subsequently analysed for cellular damage. As damage control, cells were left untreated upon OGD.

### Mitochondrial Membrane Potential Assay

The JC-1 - mitochondrial membrane potential assay kit (ab113850, Abcam) was used to determine the mitochondrial membrane polarization. In a 96-well plate 1.5 × 10^4^ N2a cells were seeded and subjected to OGD conditions for 6 h, before staining with the mitochondrial membrane potential-dependent fluorescent dye tetraethylbenzimidazolylcarbocyanine iodide JC-1 according to the manufacturer’s protocol. In brief, the cells were washed once with 100 µl/well of 1x Dilution Buffer. Next, 100 µl/well of the Working JC-1 Solution was added, and the cells were incubated for 30 min at 37 °C in the dark. The cells were then washed twice with 100 µl/well 1x Dilution Buffer solution or PBS. In the sample wells, 1 µg/ml of either WJ-MSC-sEV or *DROSHA* k.d sEV was added, and the cells were returned to a normoxic incubator. After 48 h of reoxygenation, the mitochondrial membrane potential of the cells was measured using a fluorescent plate reader (TECAN Spark 10 M, Tecan Trading AG). The excitation wavelength was set to 490 nm for green monomers (low mitochondrial membrane potential) and 535 nm for red aggregates (high mitochondrial membrane potential). The emission was measured at wavelengths of 535 nm for green monomers and 590 nm for red aggregates.

### RNA and Protein Extraction

The RNA of WJ-MSC-sEV was isolated using the Total Exosome RNA and Protein Isolation Kit (4478545, Thermo Fisher Scientific Inc.) according to the manufacturer’s instructions. RNA and protein of BV-2, MO3.13, and N2a cells were isolated using the QIAshredder and the Allprep DNA/RNA/Protein Mini Kit according to the manufacturer’s protocol (80004, Qiagen, Hilden, Germany). RNA concentration was measured using a NanoVue Plus™ spectrophotometer (Biochrom, Holliston, MA, USA). The Bicinchoninic Acid Protein Assay Kit (BCA1-1KT, Merck KGaA) was used for the determination of the total protein concentration of the cells.

### Reverse Transcription and Real-Time Polymerase Chain Reaction (PCR)

For the reverse transcription (RT) of miRNAs, the TaqMan™ Advanced miRNA cDNA Synthesis Kit (A28007, Thermo Fisher Scientific Inc.) has been used as described by the manufacturer. The reverse transcription of mRNA was done with up to 3 µg RNA using the SuperScript IV First-Strand Synthesis System (18091050, Thermo Fisher Scientific Inc.). Synthesized cDNA was stored at − 20 °C until use.

The miRNA- and gene expression were measured by real-time RT-qPCR using the primer assays listed in Table 1. For the gene expression Glyceraldehyde-3-Phosphate-Dehydrogenase (GAPDH) was used as housekeeping gene. For the miRNA expression, the results were normalized against hsa-miR-24-3p and hsa-miR-423-5p, as previously identified as most appropriate endogenous controls using a TaqMan™ Advanced miRNA Human Endogenous Controls 96-well Plate (A34643, Thermo Fisher Scientific Inc.) according to the manufacturer’s protocol. The following PCR cycling programs were run on a QuantStudio™ 7 Flex Real-Time PCR System (Thermo Fisher Scientific Inc.); for gene expression: 2 min at 50 °C, 20 s at 95 °C, followed by 45 cycles of 1 s at 95 °C and 20 s at 60 °C or miRNA expression: 20 s at 95 °C, followed by 45 cycles of 1 s at 95 °C and 20 s at 60 °C. The gene and miRNA expressions were analysed using the QuantStudio™ 7 Flex Real-Time PCR System Software.

Data were expressed as fold change relative to WJ-MSC (miRNAs), or to untreated N2a, MO3.13, or BV-2 cells (mRNAs), using the formula: Relative quantification (RQ) = 2^−ΔΔϹt^. The fold change was identified as down-regulation if RQ < 1 and upregulation if RQ > 1.

### Western Blot Analysis

The proteins were loaded onto a 4–20% gradient gel (4561094, Bio-Rad, Hercules, California, USA), separated by SDS-PAGE, transferred to nitrocellulose membranes (IB301002, Thermo Fisher Scientific Inc.), and blocked with 5% BSA (A2153, Merck KGaA) or 5% non-fat dry milk dissolved in TBST. The following proteins were analysed: Taok1 (ab197891, Abcam), p53 (30313, Cell Signaling Technology Inc., Danvers, MA, USA), Cleaved Notch1 (4147, Cell Signaling Technology Inc.), Notch1 (3608, Cell Signaling Technology Inc.), Phospho-ERK1/2 (9101, Cell Signaling Technology Inc.), and ERK1/2 (9102, Cell Signaling Technology Inc.). A horseradish peroxidase-coupled donkey anti-rabbit antibody (NA9340, Merck KGaA) was used as a secondary antibody. Binding was detected using the chemiluminescent Amersham ECL Prime Western Blotting Detection Reagent (GEHERPN2232, Cytiva Inc.) on a C-DiGit Blot Scanner (LI-COR Biosciences Inc.). Pixel summation of individual bands was performed with ImageJ Software (NIH, Bethesda, MD, USA).

### Statistical Analysis

The experiments were performed multiple independent times with at least n = 5. The data represent the mean ± SEM calculated from all assays. The statistical significance of two groups was calculated using the Mann-Whitney U-Test or the Wilcoxon signed rank test when comparing data to a normalized control. Two-way analysis of variance (ANOVA) was used to calculate statistical significance between multiple groups. Differences between groups were considered significant if adjusted p values were less than 0.05. Significance is shown with asterisks (*p < 0.05, **p < 0.01, ***p < 0.001). All analyses were done using GraphPad Prism v.9.4.1 (San Diego, CA, USA) software.

## Results

### Characterization of WJ-MSC

**Fig. 1:**
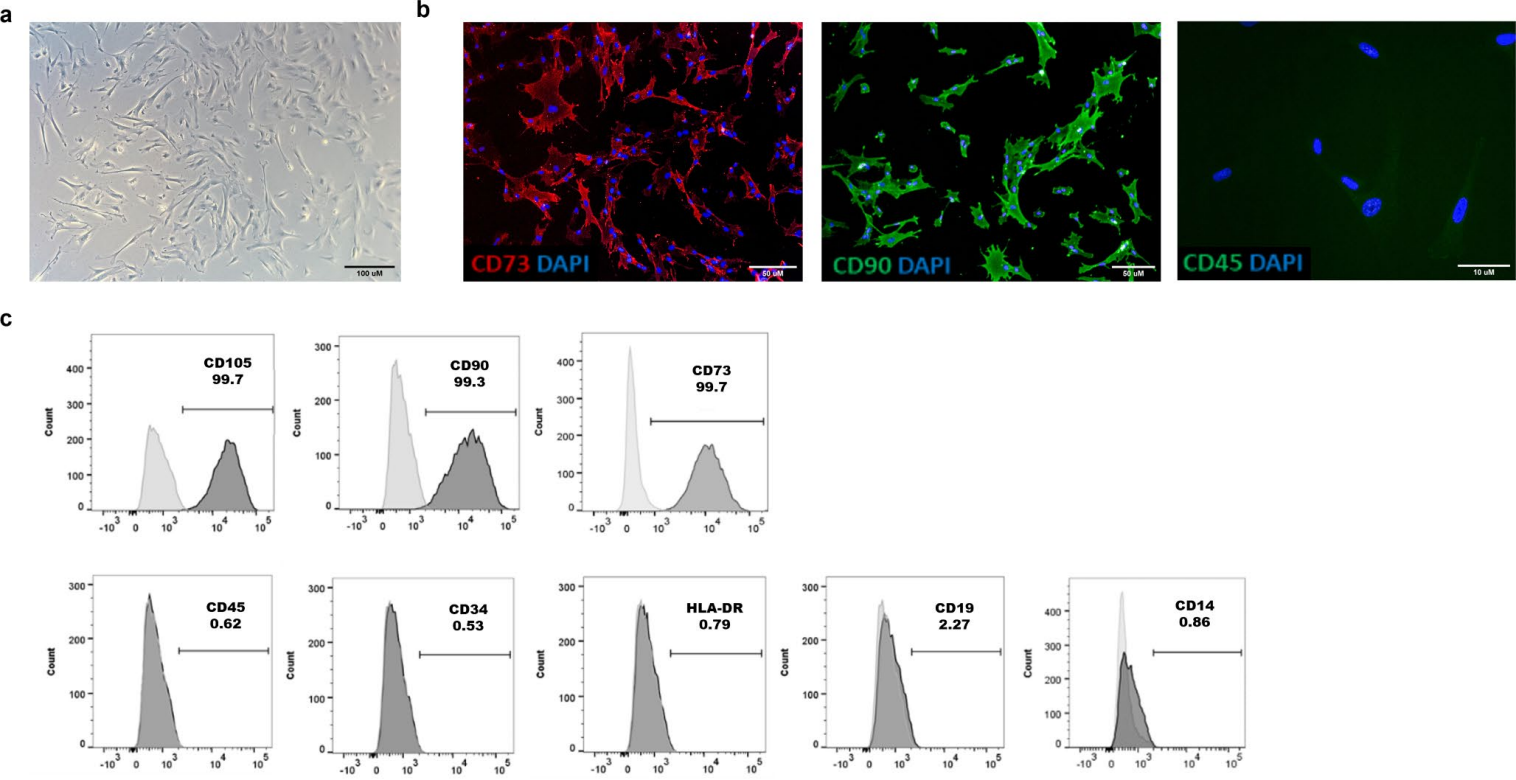
Characterization of Wharton’s jelly mesenchymal stromal cells (WJ-MSC) of three different donors. **(a)** WJ-MSC grow adherent on cell culture flasks confirmed by bright-field microscopy. **(b)** Immunocytochemistry revealed that the WJ-MSC were positive for the MSC markers 5’-nucleotidase (CD73) and Thy-1 (CD90). There was no signal for the hematopoietic marker CD45. **(c)** FACS analysis of the WJ-MSC showed the expression of MSC markers CD105, CD90, and CD73 in the samples. A broad spectrum of hematopoietic and lymphatic markers, such as CD45, CD34, HLA-DR, CD19, and CD14 were not present in the WJ-MSC samples

According to the International Society of Cellular Therapy (ISCT) [44], MSC must be plastic-adherent, express classical cell surface markers, and exhibit multipotent differentiation capacity. Our isolated WJ-MSC met all these criteria. They grew adherent on cell culture flasks (Fig. 1a) and expressed the MSC markers CD73 and CD90, whereas the hematopoietic marker CD45 was absent (Fig. 1b). In addition, > 99% of the cells were positive for MSC markers CD105, CD90, and CD73, as confirmed by flow cytometry. At the same time, the cells were negative for the cell differentiation markers CD45, CD34, CD14, CD19, and the immunogenic marker HLA-DR (Fig. 1c). We have previously shown that our WJ-MSC have the potential to differentiate into osteocytes, chondrocytes, and adipocytes [7].

### Characterization of WJ-MSC-sEV

**Fig. 2:**
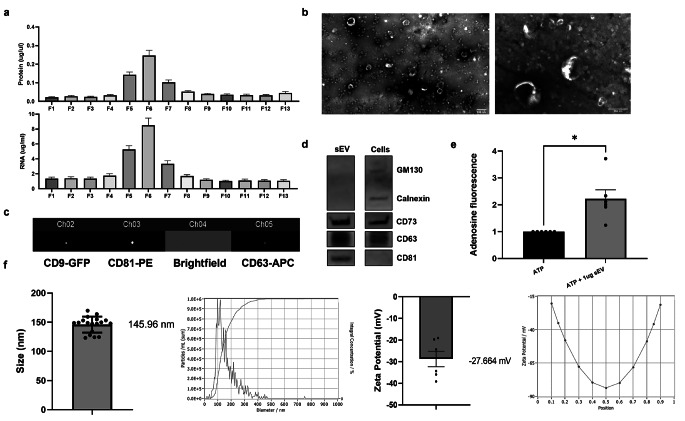
Analysis of the sEV characteristics using different techniques. **(a)** NanoVue Plus™ measurements of WJ-MSC-sEV fractions showed the highest protein and RNA content in fractions 5–7 (F5-F7), indicating the presence of sEV in these fractions. **(b)** Electron microscopy visualized the cup-shaped form of the WJ-MSC-sEV. **(c)** Imaging flow cytometry using an ImageStream Analyzer showed the presence of three tetraspanins CD9, CD81, and CD63 in the samples. **(d)** Western Blot revealed the specific sEV-protein markers CD63 and CD81 and the MSC-marker CD73 present in the WJ-MSC-sEV. The cell contamination markers GM130 and Calnexin were absent in the sEV sample. **(e)** In a fluorescence-based adenosine-production assay, the WJ-MSC-sEV showed an increase in adenosine production compared to ATP alone, displaying the integrity of the sEV by their ability to convert ATP to adenosine (Bars illustrate mean ± SEM, Wilcoxon signed rank test, * p < 0.05). **(f)** Nanoparticle tracking analysis was used to determine the size and zeta potential of the samples. The sEV from WJ-MSC had a mean size of 145.96 nm and a mean zeta potential of -27.664 mV. Representative pictures of the measurements are shown on the right of the respective graphs

Small EV are defined as round, cup-shaped membrane vesicles with a diameter of 30 to 200 nm, containing proteins and RNA, amongst other molecules [21]. After the isolation of WJ-MSC-sEV with UC, followed by SEC, we detected the highest protein and RNA contents in SEC fractions 5–7, suggesting that these represent the three main fractions containing sEV (Fig. 2a). By electron microscopy, we confirmed the cup-shaped double membrane morphology of the isolated WJ-MSC-sEV (Fig. 2b). Small EV express transmembrane and endosomal proteins; however, they do not express intracellular proteins associated with compartments other than the plasma membrane or endosomes. We confirmed WJ-MSC-sEV expression of the transmembrane tetraspanins CD63, CD81, and CD9 by imaging flow cytometry (Fig. 2c) and western blot analysis (Fig. 2d). WJ-MSC-sEV did not express the cellular contamination markers Golgi matrix protein 130 (GM130) and the integral protein of the endoplasmatic reticulum calnexin (Fig. 2d). To evaluate the integrity of the sEV cargo, it is recommended to measure the enzyme activity of the surface protein CD73 [45] that is involved in the conversion of adenosine-tri-phosphate (ATP) into adenosine. In addition, CD73 is an MSC-associated surface marker and one of the key MSC markers of the ISCT minimal criteria [44]. CD73 is expressed on the surface of MSC and susceptible to protein denaturation and loss of enzymatic activity. Thus, we measured the enzymatic acitivity of WJ-MSC-sEV membrane-bound CD73 using a fluorometric adenosine assay as an indicator for the conservation of sEV cargo integrity [45]. We showed that the addition of WJ-MSC-sEV to ATP significantly increased the production of adenosine, indicating an intact sEV cargo (Fig. 2e). In addition, the mean diameter of WJ-MSC-sEV, as measured by nanoparticle tracking analysis, was 145.96 nm (standard deviation = 13.78, minimum 123.1 nm, maximum 169.9 nm), which is within the size range known for sEV. The zeta potential indicates degree of electrostatic repulsion between particles and colloidal stability and is negative in intact sEV [46]. Our WJ-MSC-sEV had a mean zeta potential of -27.664 mV (Fig. 2f).

### WJ-MSC-sEV MiRNAs Targets *TAOK1* and *TP53*

**Fig. 3:**
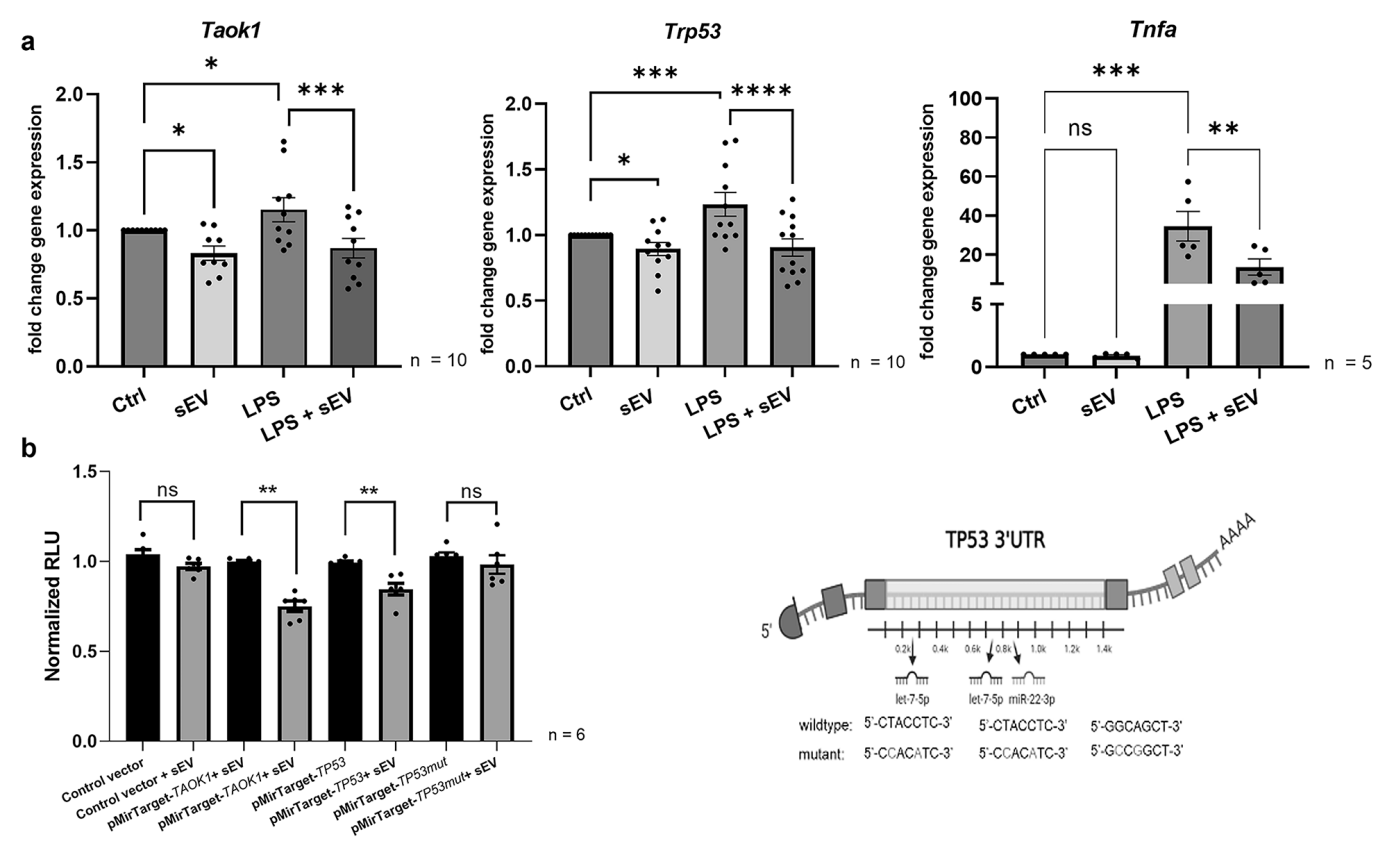
Functionality of the WJ-MSC-sEV and specifically the miRNAs in WJ-MSC-sEV. (a) BV-2 cells stimulated with 100 ng/ml LPS for 1 h displayed an increased expression of *Tnfa*. When the cells were simultaneously treated with 1 µg/ml sEV, the *Tnfa* expression was significantly reduced. Similarly, the *Trp53* and *Taok1* gene expressions increased after LPS administration and were reduced with the addition of sEV. sEV administration without LPS stimulation also showed a reduction in gene expression. (Two-way ANOVA, * p < 0.05, ** p < 0.01, *** p < 0.001, **** p < 0.0001). **(b)** A luciferase assay with vectors containing specific 3’UTR of genes involved in WMI (here TP53 and TAOK1) was used to determine the functionality of the miRNAs in WJ-MSC-sEV. The addition of sEV to HEK 293T cells transfected with a pMirTarget-TAOK and a pMirTarget-TP53 3’UTR vector reduced the luciferase signal, indicating an effect from the miRNAs. With co-transfection of the HEK293T cells with a control vector without 3’UTR or a Trp53 mutated vector (pMirTarget-TP53mut), the sEV had no effect. Schematic view of the point mutations in the mutated vector on the right side. (Bars illustrate mean ± SEM, Mann Whitney-U test, ** p < 0.01.)

*TAOK1* and *TP53* mRNA are predicted targets of miRNAs previously shown to be enriched in WJ-MSC-sEV (Suppl. Figure 1) [28]. WJ-MSC-sEV significantly reduced the transcription of *Taok1* and *Trp53* in the mouse microglia cell line BV-2 relative to control. LPS stimulation upregulated gene expression of *Taok1* and *Trp53*, which was significantly decreased by WJ-MSC-sEV (Fig. 3a). Tumor necrosis growth factor alpha (*Tnfa*) gene expression was used as a stimulation control as it is known to be upregulated during inflammation (Fig. 3a). At the protein level, the expression of Taok1 increased upon LPS challenge, which was reversed significantly by WJ-MSC-sEV. The same trend (p < 0.1) was observed for Trp53 protein expression (Suppl. Figure 2). To investigate if WJ-MSC-sEV miRNAs directly regulate the transcription of *TAOK1* and *TP53*, pMirTarget luciferase reporter vectors containing either the 3’UTR of *TP53* (pMirTarget-*TP53*) or *TAOK1* (pMirTarget-*TAOK1*) downstream of the firefly luciferase gene were transfected into HEK293T cells. The co-transfection of WJ-MSC-sEV and pMirTarget-*TP53* or pMirTarget-*TAOK1*, respectively, into HEK293T cells reduced the luciferase signal in comparison with cells transfected with the vectors in the absence of sEV (Fig. 3b). To verify that the effect of WJ-MSC-sEV was due specifically to miRNAs binding to the respective 3’UTR, the luciferase assay was repeated using either a pMirTarget luciferase vector that did not contain any 3’UTR (control vector), or a pMirTarget luciferase vector with mutated miRNA binding sites for miR-22-3p and let-7-5p in the 3’UTR of *TP53* (pMirTarget-*TP53mut*) (Fig. 3b-c). As the administration of WJ-MSC-sEV to these vectors did not decrease the luciferase signal in comparison with transfected cells in the absence of sEV, we could confirm that the effect of WJ-MSC-sEV was indeed miRNA-mediated.

### *DROSHA* Knock-Down WJ-MSC Secret sEV with Reduced MiRNA Cargo

**Fig. 4:**
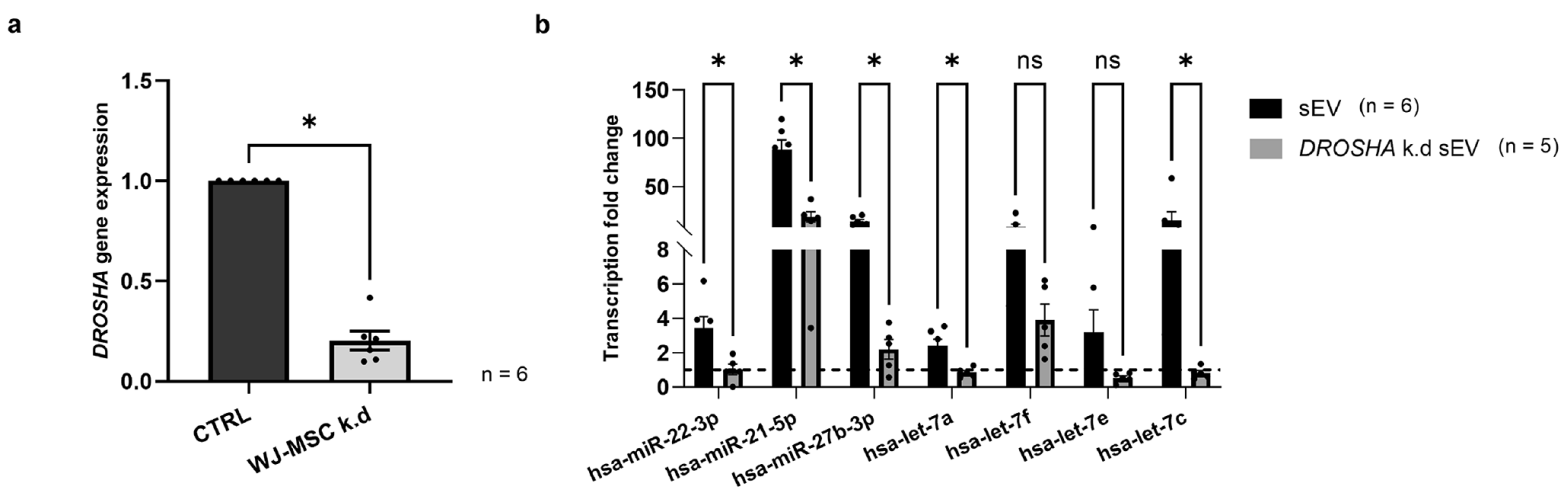
RT-qPCR analysis of the miRNA content in sEV isolated from naïve vs. *DROSHA* knock-down WJ-MSC. **(a)** The transfection of WJ-MSC with *DROSHA* siRNA decreased the *DROSHA* expression significantly with about 20-fold lower expression of *DROSHA* in the siRNA-transfected cells compared to naïve cells (Bars illustrate mean ± SEM, Wilcoxon t-test, * p < 0.05). **(b)** sEV isolated from *DROSHA* knock-down cells had significantly lower miRNAs hsa-miR-22-3p, hsa-miR-21-5p, hsa-miR-27b-3p, hsa-let-7a, and hsa-let-7c copy numbers than naïve sEV. (Bars illustrate mean ± SEM, Mann Whitney-U test, * p < 0.05)

The transfection of WJ-MSC with a silencing RNA (siRNA) against *DROSHA*, encoding a class 2 ribonuclease III enzyme which catalyses the initial processing step of miRNA biogenesis [38], resulted in a 20-fold reduction of *DROSHA* transcription in WJ-MSC (Fig. 4a). RT-qPCR confirmed that the miRNAs hsa-miR-22-3p, hsa-miR-21-5p, hsa-miR-27b-3p and members of the hsa-let-7-5p family were all upregulated in the sEV compared to the WJ-MSC. However, sEV isolated from these *DROSHA* k.d sEV contained significantly reduced levels of hsa-miR-22-3p, hsa-miR-21-5p, hsa-miR-27b-3p, hsa-let-7a and hsa-let-7c compared to naïve sEV (Fig. 4b).

### *DROSHA* Knock-Down in WJ-MSC Does Not Markedly Alter sEV Characteristics

**Fig. 5:**
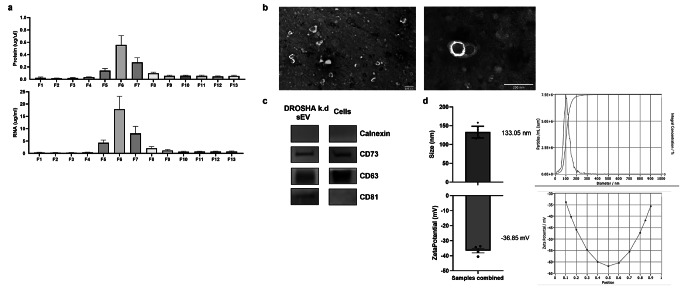
Characteristics of sEV from *DROSHA* knock-down WJ-MSC. **(a)** Measuring the different sEV fractions with a NanoVue Plus™ showed the highest protein and RNA content in fractions 5–7 (F5-F7), indicating the presence of sEV. **(b)** A cup-shaped form of sEV from *DROSHA* knock-down WJ-MSC was observed with electron microscopy (Bars illustrate mean ± SEM). **(c)** Western Blot confirmed specific sEV-protein markers, such as CD63 and CD81, and the MSC-marker CD73 in the samples. Cell contamination was excluded by the absence of Calnexin. **(d)** NanoParticle Tracking Analysis was used to determine the size and zeta potential of the samples. The *DROSHA* knock-down sEV had a mean size of 133.05 nm and a mean zeta potential of -36.85 mV. Representative pictures of the measurements are shown below the graphs (Bars illustrate mean ± SEM).

Like naïve WJ-MSC-sEV, *DROSHA* k.d sEV were retrieved in the SEC-fractions 5–7, as indicated by the high protein and RNA content (Fig. 5a). The sEV characteristics of *DROSHA* k.d sEV were confirmed by electron microscopy, western blotting, and ZetaView nanoparticle tracking analysis.

Indeed, *DROSHA* k.d sEV exhibited the characteristic cup-shaped morphology (Fig. 5b) and expressed CD81 and CD63, in the absence of calnexin (Fig. 5c). *DROSHA* k.d sEV had a mean diameter of 133.05 nm (standard deviation = 15.51, minimum 115.4 nm, maximum 158.3 nm) and a zeta potential of -36.85 mV (Fig. 5d), which is in line with known sEV size range and zeta potential.

### *DROSHA* Knock-Down sEV Fail to Inhibit Notch1 and MAPK/ERK Signalling Cascades, Negative Regulators of Oligodendrocyte Maturation

**Fig. 6:**
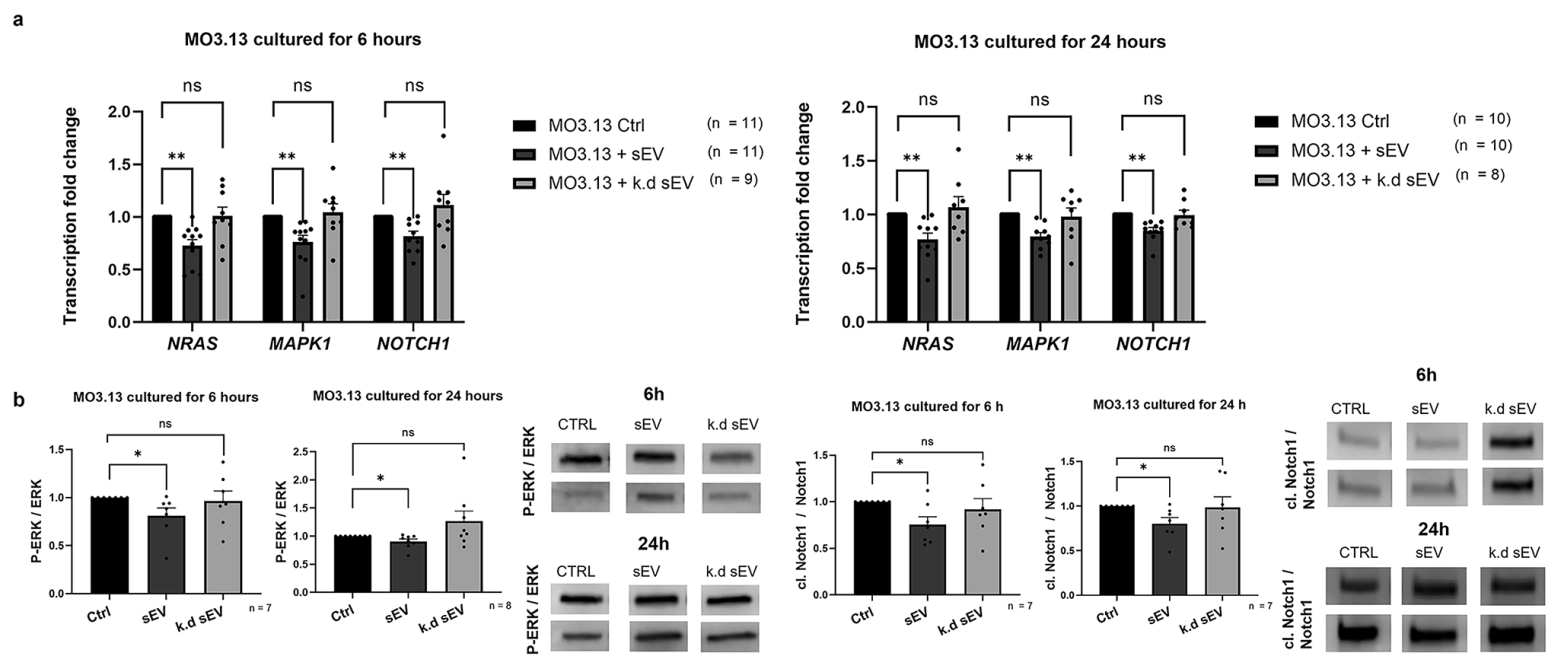
The potential to promote oligodendrocyte differentiation in sEV from naïve vs. *DROSHA* knock-down WJ-MSC. **(a) ***NRAS, MAPK1* and *NOTCH1* are known to negatively regulate oligodendrocyte differentiation. The addition of naïve WJ-MSC-sEV, but not *DROSHA* knock-down (k.d.) WJ-MSC-sEV, to MO3.13 cells results in a decrease in gene expression. **(b)** On the protein level, the same effects were observed. The phosphorylated ERK (P-ERK) ratio to ERK and cleaved Notch1 (cl. Notch1) to Notch1 was reduced when MO3.13 cells were co-cultured with naïve WJ-MSC-sEV for either 6 h, or 24 h. The effect was, however, not significant with the addition of *DROSHA* k.d WJ-MSC-sEV. (Bars illustrate mean ± SEM, Wilcoxon signed rank test * p < 0.05, ** p < 0.01)

To further assess the significance of sEV-associated miRNAs in regulating oligodendroglial maturation, we compared the effects of *DROSHA* k.d. sEV and naïve WJ-MSC-sEV on MAPK/ERK and Notch signalling pathways, known negative regulators of oligodendrocyte maturation [28, 47, 48], in the human oligodendrocyte precursor cell line MO3.13. Therefore, we assessed the functional impact of WJ-MSC-sEV miRNA on the expression of NRAS, MAPK1 and NOTCH1. The administration of naïve WJ-MSC-sEV for 6 or 24 h to MO3.13 significantly reduced the gene expression of *NRAS, MAPK1*, and *NOTCH1* relative to control cells. However, *DROSHA* k.d. sEV did not affect *NRAS, MAPK1*, and *NOTCH1* expression (Fig. 6a). The effect of naïve WJ-MSC-sEV on gene expression related to MO3.13 maturation was no longer detectable after 48 h and 72 h of co-cultivation (Suppl. Figure 3). Western blot analysis further validated the effects of naïve WJ-MSC-sEV and *DROSHA* k.d. sEV on the MAPK/ERK and Notch signalling pathways at the protein level (Fig. 6b). The stimulation of the MAPK/ERK pathway includes, amongst others, the phosphorylation of ERK1/2. Upon the treatment of MO3.13 cells with naïve WJ-MSC-sEV for 6 or 24 h, the ratio of phosphorylated ERK (P-ERK) to ERK was decreased relative to control cells, whereas there was no significant change by *DROSHA* k.d. sEV (Fig. 6b). Following activation of the Notch signalling cascade, the intracellular membrane-bound Notch transcription factor is cleaved (cl. Notch1) and translocates to the nucleus, where it triggers the transcription of Notch target genes. While the ratio cl. Notch1/Notch1 was reduced upon co-culture with naïve WJ-MSC-sEV for 6 and 24 h compared to control cells, there was no effect by *DROSHA* k.d. sEV (Fig. 6b).

### *DROSHA* Knock-Down sEV Lose their Anti-apoptotic Potential in Neuronal Cells

**Fig. 7:**
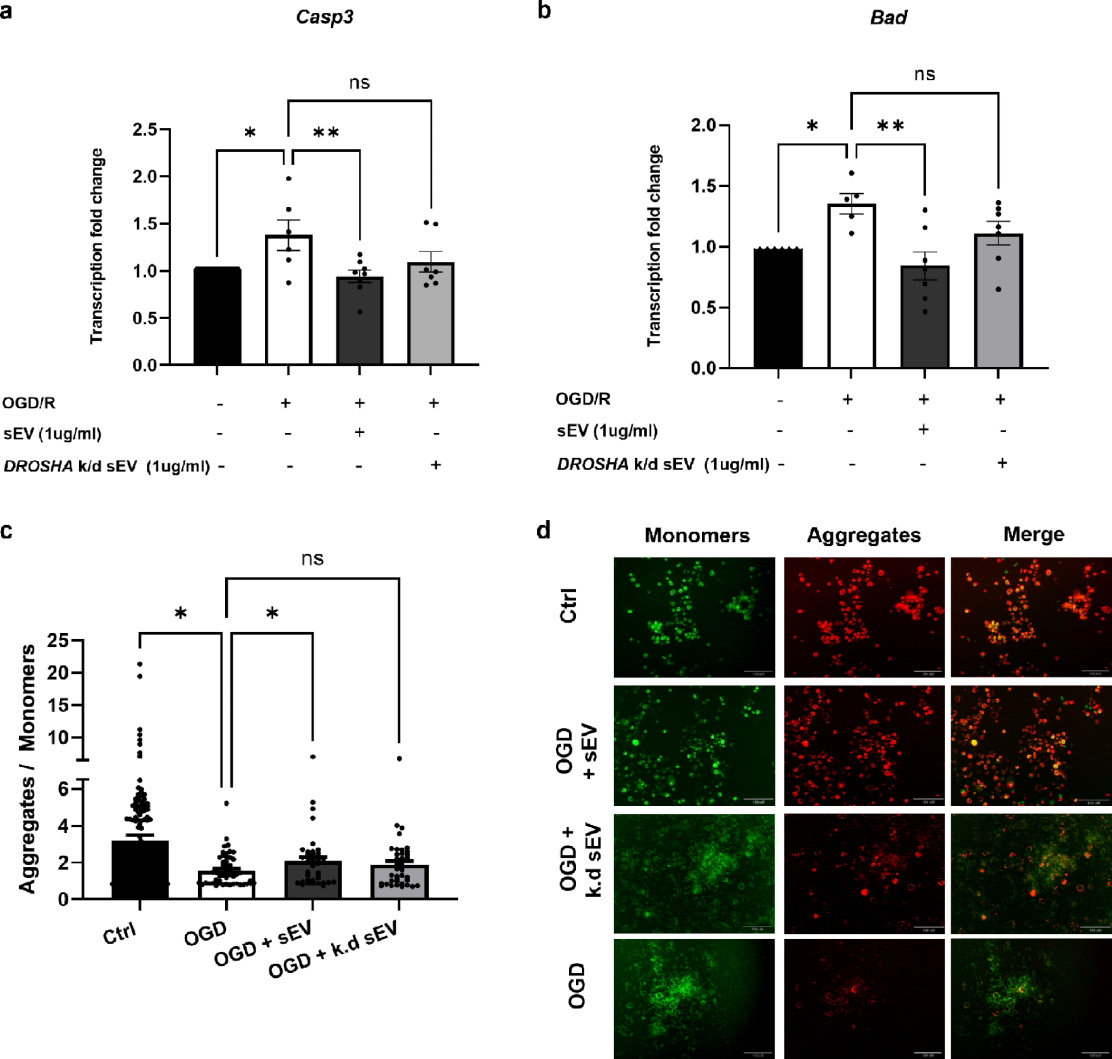
Reduction of apoptotic markers after OGD/R by naïve vs. *DROSHA* knock-down WJ-MSC-sEV. **(a, b)** After N2a cells were oxygen and glucose-deprived (OGD) for 6 h, they were returned to normal conditions and either naïve sEV, sEV from *DROSHA* knock-down (k.d.) WJ-MSC or no sEV were added. After 48 h, the apoptotic markers Caspase-3 (Casp3) and Bad were analysed. Naïve sEV, in contrast to sEV from *DROSHA *k.d WJ-MSC, significantly decreased the gene expressions compared to cells without the addition of sEV. **(c)** The mitochondrial membrane potential is reduced when cells undergo apoptosis. The membrane potential was significantly reduced in N2a cells following OGD/R, measured with a decrease in the aggregates-to-monomeres ratio of the JC-1 dye. The ratio increased significantly when the cells were treated with naïve sEV for 48 h, but not with the addition of sEV from *DROSHA* k.d WJ-MSC (Bars illustrate mean ± SEM, Two-way ANOVA, * p < 0.05, ** p < 0.01). **(d)** Microscopic images of the JC-1 assay of N2a cells.

To evaluate the therapeutic potential of sEV-associated miRNAs in HI-induced neuronal apoptosis, we compared the effects of *DROSHA* k.d sEV and naïve WJ-MSC-sEV in our previously established OGD/R in vitro model [40]. For this, the mouse neuroblastoma N2a cells were subjected to hypoxia and glucose deprivation for 6 h, followed by the addition of sEV and reoxygenation for 48 h. Naïve WJ-MSC-sEV significantly reduced the transcription of *CASP3* and *BAD*, encoding for the pro-apoptotic mediators caspase 3 (CASP3) and BCL2 associated agonist of cell death (BAD), respectively, in treated N2a cells compared to untreated cells. However, this effect was abrogated when N2a cells were treated with *DROSHA* k.d sEV (Fig. 7a, b). To analyse mitochondrial membrane potential after OGD/R, we made use of the JC-1 mitochondrial membrane assay. In intact mitochondria with high mitochondrial membrane potential, JC-1 accumulates as aggregates between the inner and outer mitochondrial membranes, emitting red to orange fluorescence. However, when the mitochondrial membrane potential is depolarized and the mitochondrial outer membrane gets permeable, JC-1 is mainly present as monomers in the cytosol leading to green colour emission [49]. Thus, open mitochondrial pores are linked to low mitochondrial membrane potential and expressed as green fluorescent monomers. In contrast, intact pores are linked to high mitochondrial membrane potential and expressed as red fluorescent aggregates. OGD/R significantly reduced the aggregates/monomers ratio, indicating increased apoptosis relative to control cells (Fig. 7c). The addition of naïve WJ-MSC-sEV for 48 h after OGD improved the mitochondrial membrane potential in N2a cells significantly compared to cells without WJ-MSC-sEV. The treated cells show a significant increase in the aggregates/monomers ratio compared to untreated cells (p = 0.0123). However, the aggregates/monomers ratio did not differ significantly upon treatment with *DROSHA* k.d sEV compared to untreated cells (p = 0.1534). The difference between cells treated with naïve sEV compared to *DROSHA* k.d sEV the effect was not significant (p = 0.7912) (Fig. 7c).

## Discussion and Conclusion

Our study provides strong evidence that mature miRNAs associated with WJ-MSC-sEV remain functional upon transfer to target cells in vitro and indicate a regulatory role in the previously observed beneficial effects of WJ-MSC-sEV in a preclinical model of WMI [7, 8]. We demonstrated that WJ-MSC-sEV miRNAs directly bind to the 3’UTR of *TP53* and *TAOK1*, genes encoding proteins involved in apoptosis and inflammation, representing key hallmarks of WMI [29–31]. The knock-down of *DROSHA* in WJ-MSC led to the secretion of sEV with significantly reduced amounts of mature miRNAs and lost the potential to prevent neuronal apoptosis and promote oligodendroglial maturation compared to naïve sEV.

We show that WJ-MSC-sEV contain significantly higher amounts of miR-21-5p, miR-22-3p, miR-27b-3p and members of the let-7 family, compared to WJ-MSC, which is in line with our previous findings [28]. The observation that the miRNA content of sEV differed strongly from their source cells is consistent with previous studies showing that miRNAs are specific for certain types of sEV [50].

As mentioned before, miR-21-5p and the let-7-5p family, both highly enriched in our WJ-MSC-sEV preparations, have essential anti-apoptotic effects by repressing *Casp3* [51, 52]. A recent study further identified miR-21-5p as a crucial factor in MSC-sEV-associated neuroprotection in neonatal rat brains upon HI injury by modulating the microglia/macrophage polarization towards an anti-inflammatory phenotype [33]. Overexpressing particular miRNAs in MSC-sEV can even further enhance putative beneficial effects [53–55]. Thus, the enrichment of EV with therapeutic miRNAs is a promising approach to drug development [56]. All findings supports the assumption that mature miRNAs are a functional unit of the beneficial effects of WJ-MSC-sEV in premature WMI.

Recently, we identified *TP53* and *TAOK1* as main predicted target genes of miRNAs highly expressed in WJ-MSC-sEV [28]. P53 and TAOK1 are actively involved in the induction of inflammation and apoptosis [29–31], both hallmarks of WMI [2, 3]. Here, we show that, in addition to *Tnfa*, WJ-MSC-sEV also decreased the transcription of *Trp53* and *Taok1* in LPS-challenged mouse microglia [2]. We further affirmed the regulatory activity of miRNA-enriched sEV on the transcription of *TP53* and *TAOK1* using 3’UTR-luciferase reporter constructs.

Today, p53 is widely accepted to activate apoptosis through both the lysosomal and the mitochondrial pathways [57]. In unstressed and untransformed cells, the levels of p53 are generally low [58]. However, in the presence of stressors, such as DNA damage and hypoxia, p53 production increases, and p53 forms a homotetrameric transcription factor inducing the transcription of target genes, driving multiple cellular processes, including apoptosis [29].

TAOK1 has been identified as a key regulator of the MAPK pathway [59] as it activates the p38 MAPK and JNK cascade upon exposure to stressors, including DNA damage, oxidative stress, and inflammatory cytokines [31]. Thereby, activated TAOK1 has been shown to induce apoptosis via the JNK pathway in a caspase-dependent way [60, 61]. Most recently, TAOK1 was identified as a positive regulator of TLR4-mediated inflammatory responses, as the overexpression of TAOK1 led to an increase in LPS-induced ERK1/2 activation and cytokine production [62]. Furthermore, TAOK1 is involved in regulating the Hippo pathway, which is crucial for controlling organ size by inhibiting cell proliferation, inducing apoptosis, and regulating the fate of stem/progenitor cells [63].

As inflammation and apoptosis are both involved in WMI [2, 3], targeting genes involved in neuroinflammation and apoptosis, such as *TP53* and *TAOK1*, as a therapeutic approach could improve WMI outcomes [8]. However, as P53 and TAOK1 also have favourable functions in tumor suppression [64] and neuronal maturation [65], respectively, the suppression of *TP53* and *TAOK1* by WJ-MSC-sEV has to be evaluated in experimental in vivo models of WMI in the future.

To test the significance of sEV miRNAs in a broader range of biological processes involved in WMI, namely oligodendroglia maturation arrest and neuronal apoptosis, we generated sEV derived from *DROSHA* knock-down WJ-MSC.

Indeed, knocking down *DROSHA* in WJ-MSC led to the production of sEV with a significantly decreased miRNA expression. We decided to knock-down *DROSHA* instead of *DICER1*, because the loss of *DICER1* impairs cell survival [66]. In multiple cell types, including MSC, the loss of Dicer1 inhibits cell proliferation and promotes premature senescence [67]. Furthermore, as Drosha initiates miRNA processing in the nucleus [38], its knock-down prevents the export of the pre-miRNAs into the cytoplasm via the canonical pathway, allowing only very few miRNAs to become mature.

Premature WMI occurs at a time when oligodendrocyte precursors that are particularly vulnerable to damage caused by oxidative stress and free radicals, are the predominant cells of the oligodendrocyte linage, leading to an oligodendrocyte maturation arrest. The Notch and MAPK/ERK signalling pathways have been identified as negative regulators of oligodendrocyte maturation [28, 47, 48]. In line with our recent study, we found that WJ-MSC-sEV drive the maturation of oligodendroglial precursor cells by inhibiting the activation of MAPK/ERK and the Notch1 cascade and the transcription of genes encoding proteins involved in these pathways, namely *NRAS, MAPK1* and *NOTCH1* [28]. Interestingly, we observed that sEV derived from *DROSHA* knock-down WJ-MSC had lost their potential to reduce the transcription of *NRAS, MAPK1* and *NOTCH1*, indicating that sEV miRNAs are involved in the induction of oligodendrocyte maturation by repressing MAPK/ERK and Notch1 signalling. We confirmed the expression of several sEV miRNAs, such as hsa-miR-22-3p, hsa-miR-21-5p, hsa-miR-27b-3p, and members of the let-7 family, predicted to target genes involved in MAPK1 and Notch1 signalling. These miRNAs were significantly lower in *DROSHA* knock-down sEV (Fig. 4b), indicating that the reduced effect of the *DROSHA* knock-down sEV on the MAPK1 and Notch1 signalling is – at least partially - due to the reduction of miRNAs.

During the intrinsic apoptosis pathway, pro-apoptotic stressors, such as OGD/R, trigger the opening of mitochondrial permeability transition pores, resulting in the loss of mitochondrial transmembrane potential and, thus, the initiation of the apoptotic cascade [68]. Together with *Casp3* activation, the loss of mitochondrial transmembrane potential occurs as secondary damage in premature WMI and accounts for a major part of neuronal cell death upon exposure to HI [69]. Our study demonstrated that OGD/R initiated the mitochondrial apoptosis cascade by the depolarization of the mitochondrial membrane potential in neuronal cells, which was prevented by WJ-MSC-sEV. Furthermore, we showed that OGD/R increased neuronal expression of *Casp3* and its downstream target *Bad*. WJ-MSC-sEV impeded with OGD/R-mediated upregulation of *Casp3* and *Bad*, which is in accordance with our previous findings [40]. Our study, in contrast, revealed that *DROSHA* knock-down sEV have neither reverted mitochondrial membrane depolarization nor reduced the gene expression of the apoptotic genes *Casp3* and *Bad* upon OGD/R. This observation provides robust evidence for the significant involvement of mature miRNAs associated with WJ-MSC-sEV, such as miR-21-5p and the let-7-5p family, in blocking the mitochondrial cascade of apoptosis –at least partially – by repressing *Casp3* [51, 52]. However, the difference in mitochondrial membrane potential is not significant between cells treated with naïve sEV versus *DROSHA* knock-down sEV. This indicates that the *DROSHA* knock-down sEV may still have some effect on their target cells.

Apart from miRNA processing, Drosha has been shown to play a role in many other cellular processes, such as post-transcriptional control of mRNA stability, cell fate specification and differentiation, and antiviral activity [70]. Thus, the deficiency of Drosha could imply changes in cell state, possibly including alterations in the composition of the secreted sEV. However, we did not observe any marked changes in the morphology, size, zeta potential and endosomal marker expression in sEV derived from *DROSHA* knock-down WJ-MSC relative to naïve sEV. Hence, we considered it unlikely that the *DROSHA* knock-down negatively affected WJ-MSC physiology during the short culture time needed for sEV generation. A minority of miRNAs are processed in a Drosha-independent way. Our analysis does not represent the potentially beneficial function of this subset of miRNAs [36]. The functionality of the most promising therapeutic sEV miRNA in WMI should be further validated in future experiments by knocking them down individually.

Our data strongly indicate that miRNAs are functional units of WJ-MSC-sEV’ beneficial effects in premature WMI. Besides miRNA, the cargo of MSC-sEV comprises many other signalling molecules, and it is unlikely that the regulatory role of WJ-MSC is solely attributed to miRNAs. Several proteins, including transcription factors, and translational regulators derived from MSC-EV have been suggested to be involved in regulating recipient cells [71]. In the future, it is essential also to consider transcriptomic and proteomic analyses of WJ-MSC-sEV.

To conclude, we identified miRNAs of WJ-MSC-sEV as a key contributor to preventing mitochondrial-dependent apoptosis and enhancing oligodendrocyte maturation in vitro. Our results suggest the regulatory role of sEV miRNA cargo in the therapeutic effects of MSC-sEV in preclinical WMI models. However, our study’s findings are based on in vitro experiments, reflecting a potential limitation of our work. Data obtained by in vitro experimentations cannot be directly applied to the in vivo situation, as in vitro studies fail to mimic the complexity and conditions of a living organism. Hence the effect of WJ-MSC-sEV might be different in the living system. However, we are convinced that the in vitro models can strongly reflect the functionality of WJ-MSC-sEV miRNAs upon transfer into recipient cells. Nevertheless, to complete our understanding of WJ-MSC-sEV miRNAs as functional therapeutic mediators, their regulatory potential has to be confirmed in an in vivo animal model of premature WMI.

### Electronic Supplementary Material

Below is the link to the electronic supplementary material.

Supplementary Material 1

Supplementary Material 2

Supplementary Material 3

Supplementary Material 4

## Data Availability

The datasets generated during and/or analysed during the current study are available from the corresponding author upon reasonable request.
