# Supplementary material for: MicroRNA Cargo in Wharton’s Jelly MSC Small Extracellular Vesicles: Key Functionality to In Vitro Prevention and Treatment of Premature White Matter Injury
Source: Stem Cell Rev Rep. 2023 Jul 31;19(7):2447–64. doi: 10.1007/s12015-023-10595-1 (PMC10579138; doi:10.1007/s12015-023-10595-1)
Supplement: Supplementary file 1 — Supplementary Material 1 [file 12015_2023_10595_MOESM1_ESM.docx]

**Supplementary Table 1:** Antibody Dilutions of Antibodies used for FACS

| **Antibody** | **Order Details** | **Dilution used for FACS** |
| --- | --- | --- |
| CD105-APC | BD Biosciences, Cat. No. 550256 | 1:50 |
| CD90-FITC | Acris, Cat. No. SM1170F | 1:10 |
| CD73-FITC | BD Pharmingen, Cat. No. 550256 | 1:10 |
| CD45-FITC | BD Pharmingen, Cat. No. 555482 | 1:5 |
| CD34-FITC | BD Pharmingen, Cat. No. 555821 | 1:5 |
| CD19 | Millipore, FCMAB184F, clone HD37 | 1:10 |
| CD14-FITC | Chemicon, Cat. No. MAB1219F | 1:10 |
| HLA-DR-FITC | BD Pharmingen, Cat. No. 555811 | 1:10 |
| 2^nd^ IgG-Alexa Fluor 488 | Invitrogen, Cat. No. A-11001 | 1:200 |

**Supplementary Table 2** Program cycle used to make point mutations in the TP53 vector

| **Segment** | **Cycles** | **Temperature** | **Time** |
| --- | --- | --- | --- |
| 1 | 1 | 95 °C | 2 min |
| 2 | 18 | 95 °C | 20 sec |
|  |  | 60 °C | 10 sec |
|  |  | 68 °C | 30 sec/kb of plasmid length |
| 3 | 1 | 68 °C | 5 min |
