# Supplementary figures and images for: MicroRNA Cargo in Wharton’s Jelly MSC Small Extracellular Vesicles: Key Functionality to In Vitro Prevention and Treatment of Premature White Matter Injury
Source: Stem Cell Rev Rep. 2023 Jul 31;19(7):2447–64. doi: 10.1007/s12015-023-10595-1 (PMC10579138; doi:10.1007/s12015-023-10595-1)

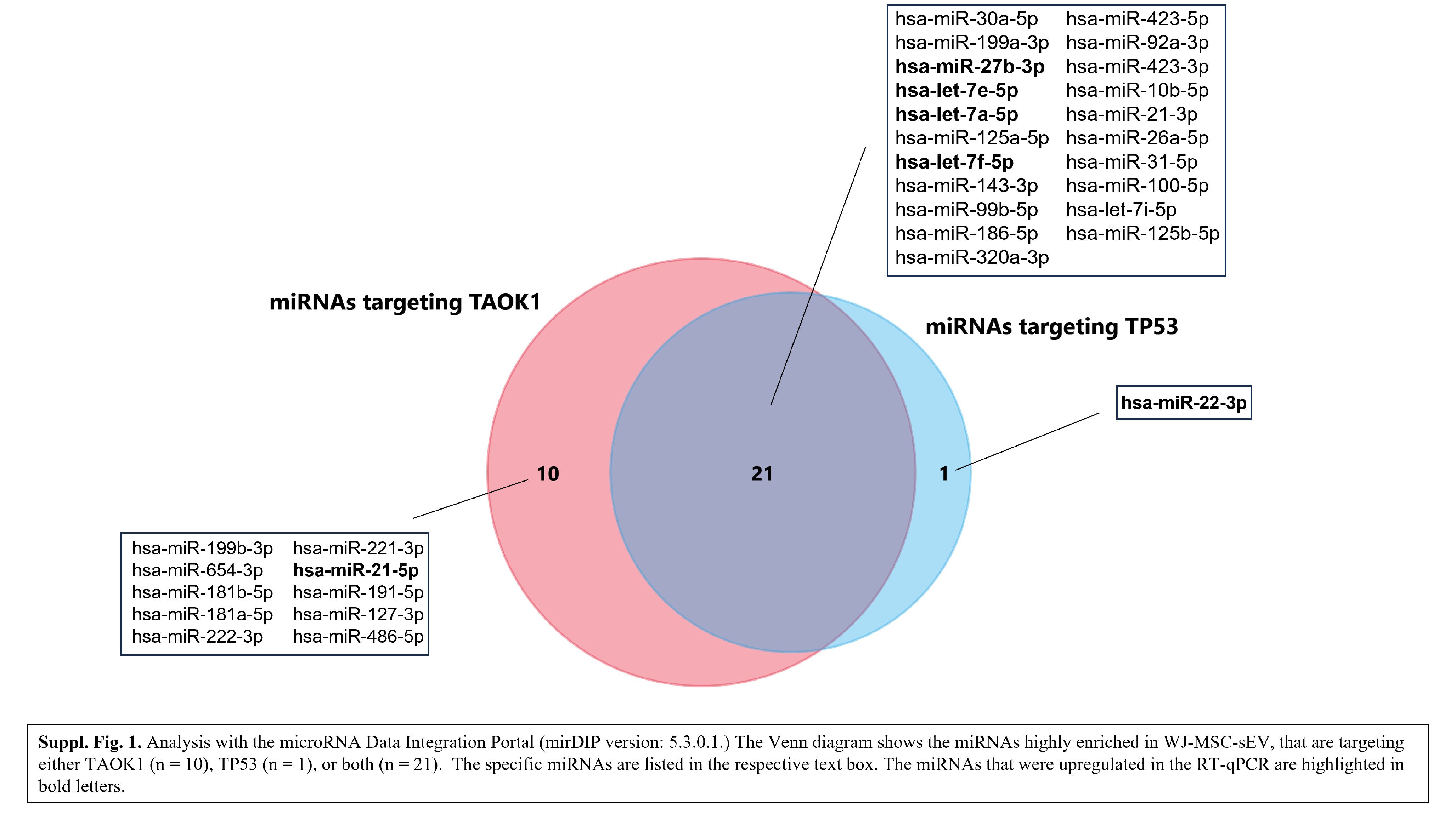

Supplement: Supplementary file 2 — Supplementary Material 2 [file 12015_2023_10595_MOESM2_ESM.tif]

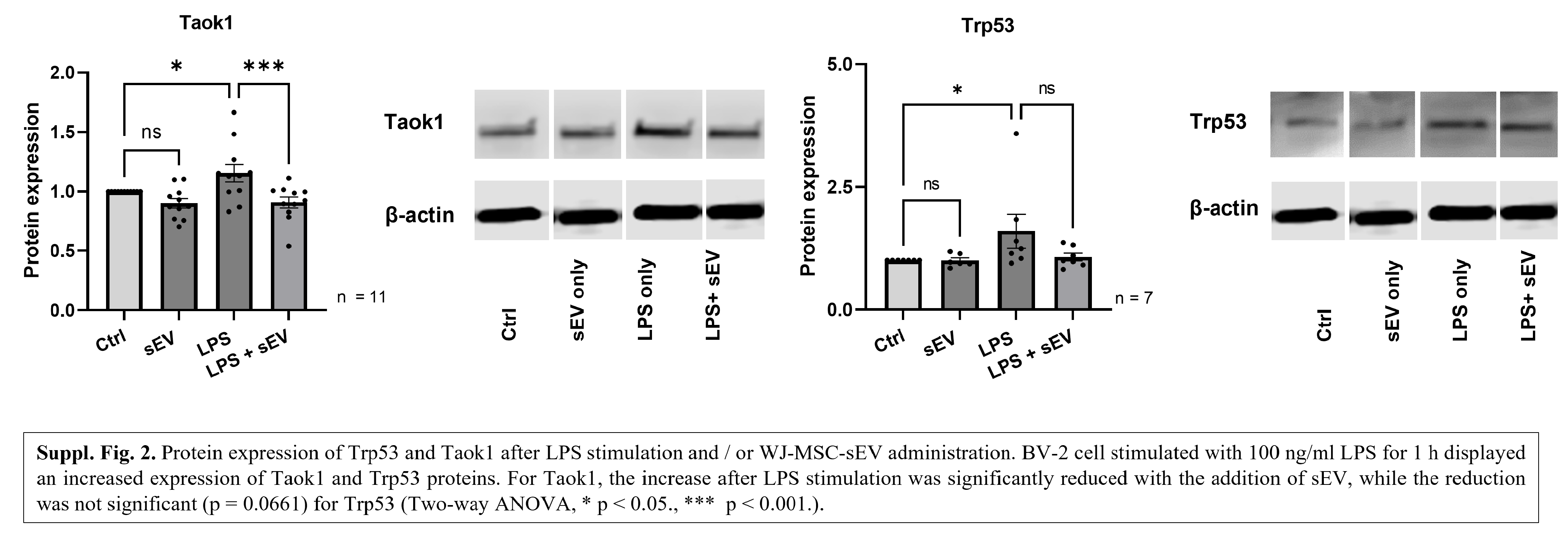

Supplement: Supplementary file 3 — Supplementary Material 3 [file 12015_2023_10595_MOESM3_ESM.tif]

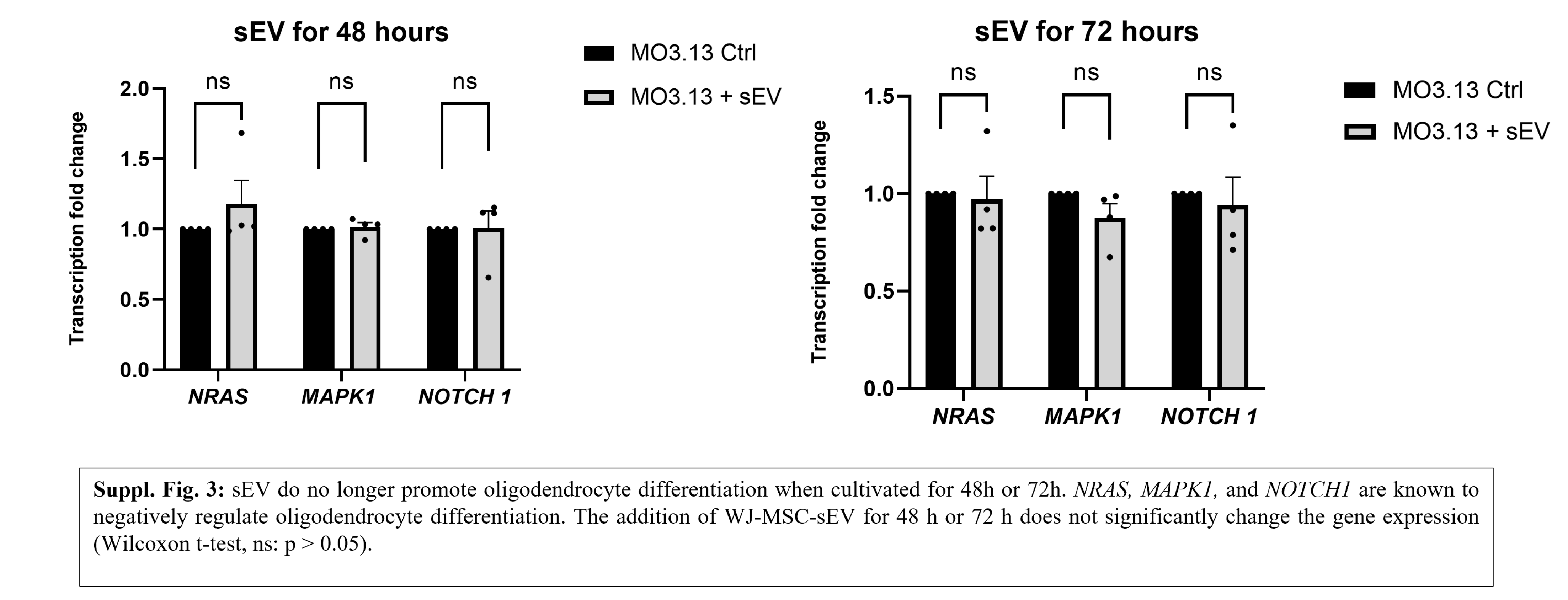

Supplement: Supplementary file 4 — Supplementary Material 4 [file 12015_2023_10595_MOESM4_ESM.tif]
